# Short-term therapeutic efficacy analysis of drug-coated balloon combined with chocolate balloon for the treatment of femoropopliteal artery lesions

**DOI:** 10.3389/fsurg.2025.1419127

**Published:** 2025-03-28

**Authors:** Rong Zhang, Chao Yun Jiang, Tian Hong Cai, Jiang Feng He, Kai Chen, Teng Hui Zhan

**Affiliations:** Department of Vascular Surgery & Interventional Treatment, Fujian Maternity and Child Health Hospital, College of Clinical Medicine for Obstetrics & Gynecology and Pediatrics, Fujian Medical University, Fuzhou, China

**Keywords:** DCB, chocolate balloon, femoropopliteal artery lesion, short-term efficacy, treatment

## Abstract

**Background:**

To report our experience of short-term results of drug-coated balloon (DCB) combined with chocolate balloon in the treatment of femoropopliteal artery lesions.

**Methods:**

From June 2021 to December 2022, patients with femoropopliteal artery lesions (Rutherford classification 2–6) who underwent DCB combined with Chocolate PTA balloon catheter treatment were included. Clinical data of the patients were collected, and follow-up was conducted at 3, 6, and 12 months. The primary patency rate and the freedom from clinically-driven target lesion revascularization (f-TLR) rate were calculated by Kaplan–Meier survival curves.

**Results:**

This study included a total of 43 patients (mean age 72.84 ± 10.19 years, male proportion 67.4%) with 47 lesions. Among them, 17 lesions (36.2%) presented severe stenosis with an average lesion length of 110.41 ± 47.67 mm. Thirty lesions (63.8%) were identified as chronic total occlusions (CTO), with an average occlusion length of 104.13 ± 61.12 mm. The Kaplan–Meier survival curve estimated a primary patency rate of 87.2% at 6 months and 78.7% at 12 months. The f-TLR rate at 12 months was 85.1%, estimated by Kaplan–Meier survival curve. The mean ankle-brachial index (ABI) increased from 0.53 ± 0.12 before the surgery to 0.87 ± 0.12 at 12 months postoperatively, and this difference was statistically significant (*p* < 0.001). A total of 91.5% of patients (43/47) showed a decrease in Rutherford classification at 12 months postoperatively. The proportion of patients with Rutherford class 4–6 decreased from 70.2% (33/47) preoperatively to 4.3% (2/47) at 12 months postoperatively, and this difference was statistically significant (*p* < 0.001). Among the limbs, 34 (72.3%) experienced dissection during the surgery, with 29 cases classified as type B or lower dissection and 5 cases classified as type C or higher (severe dissection) (10.6%). Two limbs (4.3%) required the use of salvage stents. There were no procedure- or device-related deaths within the 12-month period. Twelve limbs (25.5%) underwent minor amputations (toe amputations).

**Conclusion:**

The combination of DCB and chocolate balloon angioplasty has achieved satisfactory patency rates and f-TLR results in 1-year follow-up for the treatment of femoropopliteal artery lesions. However, further confirmation of these findings is needed through multicenter data and long-term follow-up results.

## Introduction

Peripheral artery disease (PAD) can lead to symptoms such as intermittent claudication, ischemic rest pain, ulcer, or gangrene, seriously affecting the quality of life of patients (1). With the aging population, the incidence of PAD has been steadily increasing worldwide. It is estimated that there are currently around 240 million PAD patients in the world (2), with 80%–90% of PAD patients aged 60 and above having femoropopliteal artery lesions (3).

Percutaneous transluminal angioplasty (PTA) is currently the first-line treatment for femoropopliteal artery lesions (4), and plain old balloon angioplasty (POBA) is prone to complications such as dissection due to factors like vascular calcification and long-segment lesions. Although it can be solved by placing a salvage stent, the issue of in-stent restenosis (ISR) that occurs after stent placement is even more challenging. It has been reported that the incidence of ISR within one year after POBA in patients with femoropopliteal artery lesions can be as high as 60% (5). The main cause of ISR after PTA is excessive intimal hyperplasia, and the application of drug-coated balloon (DCB) can improve the efficacy of PTA (6). DCBs utilize a matrix coating method to uniformly apply anti-proliferative drugs (paclitaxel, rapamycin, etc.) onto the surface of the balloon. These drugs inhibit cell division by binding to microtubule proteins, thereby suppressing neointimal hyperplasia and improving post-procedural patency rates (7, 8). Although DCB have achieved favorable therapeutic outcomes in femoropopliteal artery lesions, the rate of salvage stent placement is not low due to the flow-limiting dissection after PTA. A study revealed that the incidence of severe flow-limiting dissections (Type C–F) after superficial femoral artery (SFA) PTA was 42%, requiring subsequent interventions and salvage stent placement in the majority of cases (9). Therefore, in order to minimize the occurrence of dissections and ensure non-metallic interventions while fully leveraging the advantages of DCB, it is crucial to optimize balloon angioplasty to achieve adequate vessel preparation prior to DCB deployment. The Chocolate PTA balloon catheter is a semi-compliant balloon that incorporates nickel-titanium wire constraints on its surface. This allows the expanded balloon to be divided into independently expanding segments, resulting in a “pillow effect” that increases the contact surface area between the balloon and the vessel. The design of this balloon aims to disperse the forces associated with angioplasty along this increased contact surface, reducing vascular wall damage and minimizing the occurrence of dissections to the greatest extent possible (10). In 2022, a Japanese study showed a 4.2% incidence of severe dissection and a 2.1% salvage stenting rate after PTA using a chocolate balloon in femoropopliteal artery lesions (11). At present, there is limited literature on the use of DCB combined with chocolate balloon for the treatment of femoropopliteal artery lesions. In this study, we collected and analyzed the clinical data from our department regarding the treatment of femoropopliteal artery lesions using this combination of DCB and chocolate balloons, and we present the short-term results.

## Method

### Patients

Patients with femoropopliteal artery disease who were admitted to Fujian Maternity and Child Health Hospital from June 2021 to December 2022 were selected as the research subjects. Inclusion criteria: (1) age >18 years old; (2) Rutherford grade 2–6; (3) superficial femoral artery and/or popliteal artery severe stenosis (≥70%) or occlusive lesions; (4) There is at least one unobstructed distal outflow tract under the knee; (5) Sign the informed consent. Exclusion criteria: (1) Conditions that can cause acute lower limb ischemia, such as lower extremity arterial thrombosis, lower extremity arterial embolism, and thromboangiitis obliterans; (2) Use of medications or participation in other clinical trials within the past 3 months that could interfere with the results of this study; (3) Glomerular filtration rate <30 ml/min; (4) Allergy to medications such as aspirin, clopidogrel, heparin, iodinated contrast agents, and paclitaxel; (5) In-stent restenosis(ISR); (6)) combined with severe coronary heart disease, hepatic insufficiency, pulmonary disease, or other conditions that make the patient unsuitable for surgery; (7) Anticipated survival of less than six months; (8) coagulation dysfunction, bleeding tendency; (9) Follow-up period less than 3 months.

### Intervention

All patients were placed in the supine position, and the arterial sheath was inserted after successful puncture of the femoral artery. After the sheath placement, systemic heparinization was performed (80–100 U/kg). If antegrade access was difficult, retrograde access was utilized. The retrograde access was performed under ultrasound guidance, primarily through the posterior tibial artery or anterior tibial artery, depending on the vessel diameter and quality. After successful puncture with a 4F micropuncture set, crossed the occluded segment and performed balloon angioplasty follow the same protocol as described for antegrade access. Following the passage of a 0.014-inch or 0.018-inch guidewire through the target lesion, sequential pre-dilatation with a regular balloon was performed for vessel preparation. Pre-dilatation was performed using a non-compliant balloon with the diameter sized 0.5–1.0 mm smaller than the reference vessel diameter. The balloon length was selected to match the lesion length. For heavily calcified lesions, high-pressure balloon inflation (>14 atm) was performed. Once approaching the diameter of the target lesion vessel, the regular balloon was replaced with a chocolate balloon for PTA. The target vessel diameter was measured using quantitative vascular angiography (QVA) at the reference vessel segments proximal and distal to the lesion. The mean of these measurements was used as the reference vessel diameter for device sizing. The diameter of the chocolate balloon was selected based on the diameter of the target lesion vessel (1:1 ratio). It was inflated to half of the working pressure and held for 30 s, followed by slow inflation to the working pressure and held for 180 s. Subsequently, a DCB (3 mg/mm^2^, Medtronic Inc.) was used for a 180-s dilation. The diameter of the DCB balloon was chosen based on the diameter of the target lesion vessel (1:1 ratio). If residual stenosis >30% or dissections of type D or higher were observed during follow-up angiography, a salvage stent was implanted.

### Medical treatment

All patients received preoperative oral antiplatelet medications (aspirin 100 mg/day and clopidogrel 75 mg/day) for at least 3 days. After the procedure, dual antiplatelet therapy was continued for 6 months, followed by a switch to long-term monotherapy with a single antiplatelet medication.

### Follow-up and study endpoints

Patients underwent outpatient follow-up examinations every 3 months within the first year after surgery, followed by biannual examinations. The follow-up assessments included Rutherford classification, lower extremity arterial ultrasound, ankle-brachial index, assessment of wound healing status (for patients with tissue loss), and evaluation of any adverse events. The follow-up period was concluded on April 30, 2023.

The primary endpoint was patency, defined as the absence of clinically driven-target lesion revascularization (CD-TLR) and no target lesion restenosis (>50%) confirmed by Doppler ultrasound (peak systolic velocity ratio <2.4) or angiography. CD-TLR was defined as the need for repeat intervention on the target lesion due to symptomatic recurrence, a decrease in ankle-brachial index by >0.15 or a decrease of ≥20% compared to the immediate postoperative value during follow-up.

Secondary endpoints: (1) Freedom from CD-TLR rate (f-TLR): Defined as the absence of repeat percutaneous or surgical revascularization of the target lesion or within a 5 mm border proximal or distal to the lesion; (2) Safety endpoints: Including amputation rate, target vessel acute thrombosis, and mortality; (3) Salvage stent placement rate; (4) Technical success: Successful establishment of access, deployment of drug-coated balloon within the target lesion, and residual stenosis <30%.

### Statistical methods

Statistical analysis was performed using SPSS 23.0 software. Statistical analysis was performed using SPSS 23.0 software. Normally distributed continuous data are presented as mean ± standard deviation, while categorical data are presented as frequency and percentage. Group comparisons were conducted using the chi-square test or Fisher's exact test. Cumulative patency rates and f-TLR were calculated using Kaplan–Meier survival curves. A significance level of *P* < 0.05 was considered statistically significant.

## Result

### Demographics and lesion characteristics

This study included patients predominantly in their early 70s, with a male majority. The mean follow-up time exceeded 1 year post-operation. The majority of lesions were classified as *de novo* stenosis, involved both the femoral and popliteal arteries, and had chronic total occlusion (CTO) with an average occlusion length of more than 10 cm (87.2%, 42.6%, and 63.8%, respectively). Most patients presented with advanced disease stages, as reflected by their Rutherford classification and ankle-brachial index values Baseline clinical characteristics are presented in Table 1.

**Table 1:** Baseline characteristics of 43 patients/47 limbs included in this series.

Variables	43 patients/47 limbs
Age, years mean	72.84 ± 10.19
Male gender	67.4 (29)
Diabetes	51.2 (22)
Hypertension requiring medications	65.1 (28)
Hyperlipidemia requiring medications	53.5 (23)
BMI	22.52 ± 4.07
Prior stroke	16.3 (7)
Prior MI	2.3 (1)
History of CAD	25.6 (11)
Smoker	30.2 (13)
Renal insufficiency(GFR <60 ml/min/1.73 m^2^)	37.2 (16)
ABI	0.53 ± 0.12
Baseline mean Rutherford class	4.19 ± 0.96
2—Moderate claudication	6.4 (3)
3—Severe claudication	21.3 (10)
4—Ischemic rest pain	29.8 (14)
5—Minor tissue loss	40.4 (19)
6—Major tissue loss	2.1 (1)

Values are mean ± stand deviation (SD) or % (*n*).

BMI, body mass index; MI, myocardial infarction; CAD, coronary artery disease; GFR, glomerular filtration rate; ABI, ankle-brachial index.

In this study, 6 cases (12.8%) were classified as restenosis, and 41 cases (87.2%) were classified as *de novo* stenosis. Among the lesions, 13 cases (27.7%) solely involved the femoral artery, 14 cases (29.8%) solely involved the popliteal artery, and 20 cases (42.6%) involved both the femoral and popliteal arteries. Among the limbs, 17 (36.2%) exhibited severe stenosis, with an average lesion length of 110.41 ± 47.67 mm. Thirty limbs (63.8%) had chronic total occlusion (CTO) lesions, with an average occlusion length of 104.13 ± 61.12 mm. Seven limbs (14.9%) had CTO lengths exceeding 150 mm. The anatomical and preoperative lesion characteristics of the target vessels are presented in Table 2.

**Table 2:** Baseline and procedural lesion characteristics.

Variables	47 limbs
Lesion type	
*de novo*	87.2 (41)
Restenosis	12.8 (6)
Lesion location	
SFA	27.7 (13)
PA	29.8 (14)
SFA and PA	42.6 (20)
Outflow vessels number	
0	0
1	12.8 (6)
≥2	87.2 (41)
Calcium score	
CS0	4.3 (2)
CS1	17.0 (8)
CS2	27.7 (13)
CS3	29.8 (14)
CS4	21.3 (10)
Stenosis	
Limbs	36.2 (17)
Mean length (mm)	110.41 ± 47.67
Mean diameter reduction (%)	81.18 ± 6.00
CTO	
Limbs	63.8 (30)
Mean length (mm)	104.13 ± 61.12
Occlusion >150 mm	14.9 (7)

Values are mean ± stand deviation (SD) or % (*n*).

SFA, superficial femoral artery; PA, popliteal artery; CS, calcium score; CTO, chronic total occlusion.

### Efficiency outcomes

**Figure 1:**
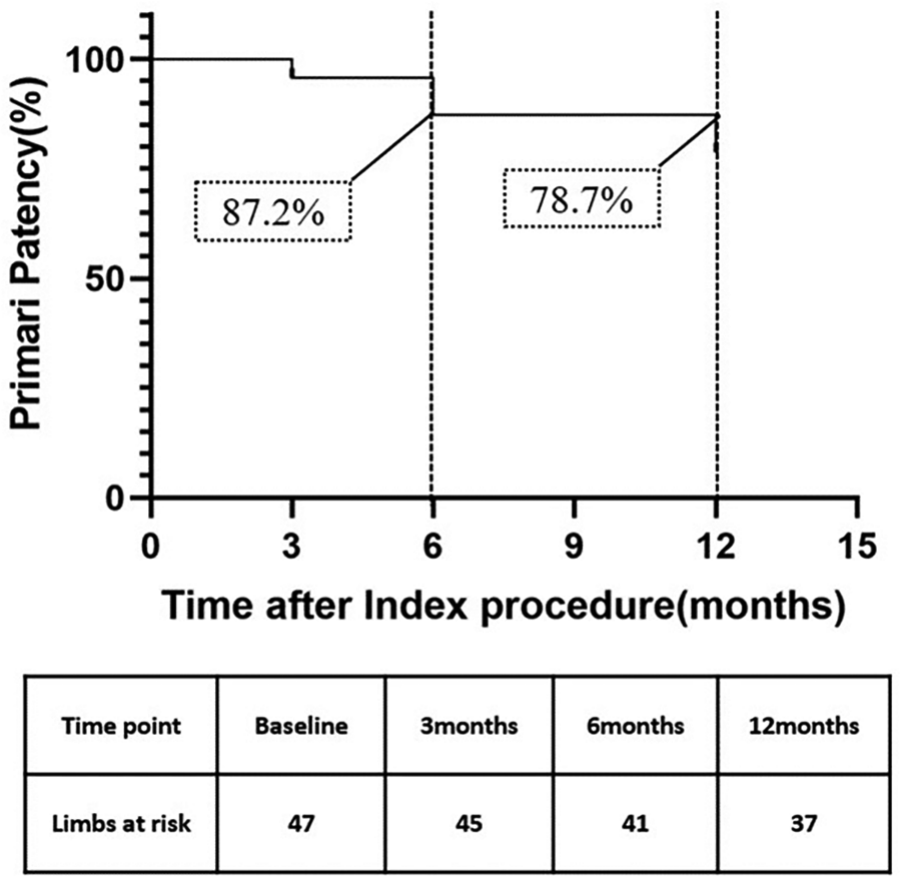
Kaplan–Meier curves of primary patency.

**Figure 2:**
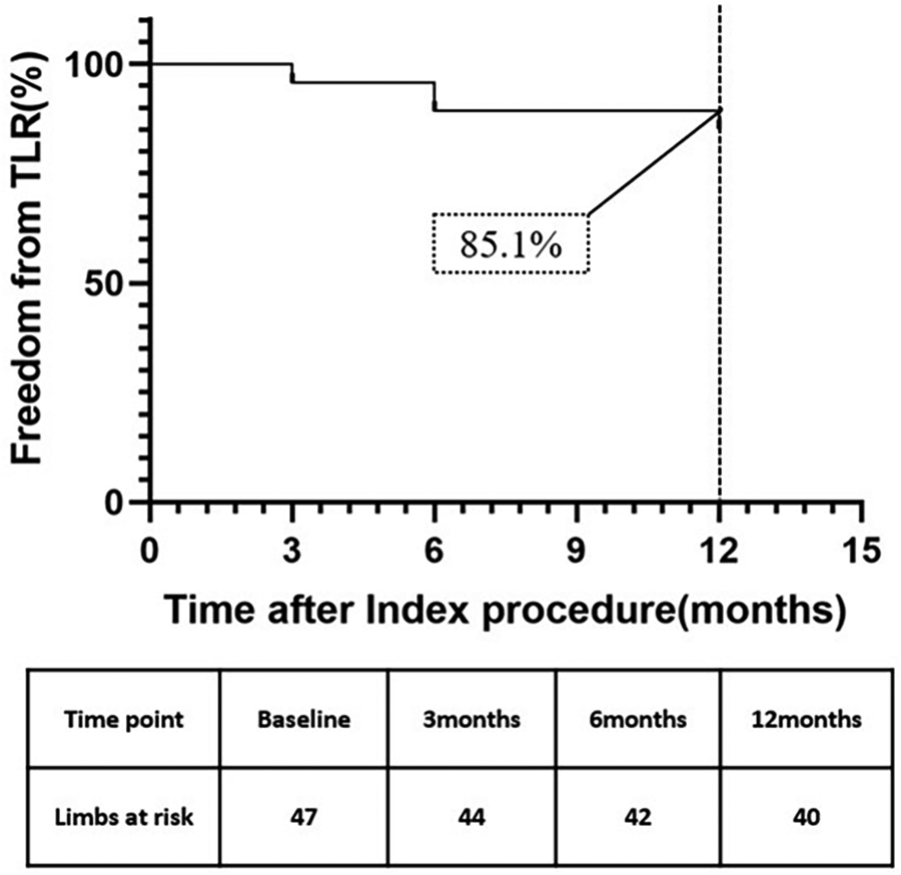
Kaplan–Meier curves of freedom from TLR.

The Kaplan–Meier survival curve estimated primary patency rate was 87.2% at 6 months and 78.7% at 12 months (Figure 1). According to Kaplan–Meier survival curve estimation, the f-TLR rate at 12 months was 85.1% (Figure 2).

**Figure 3:**
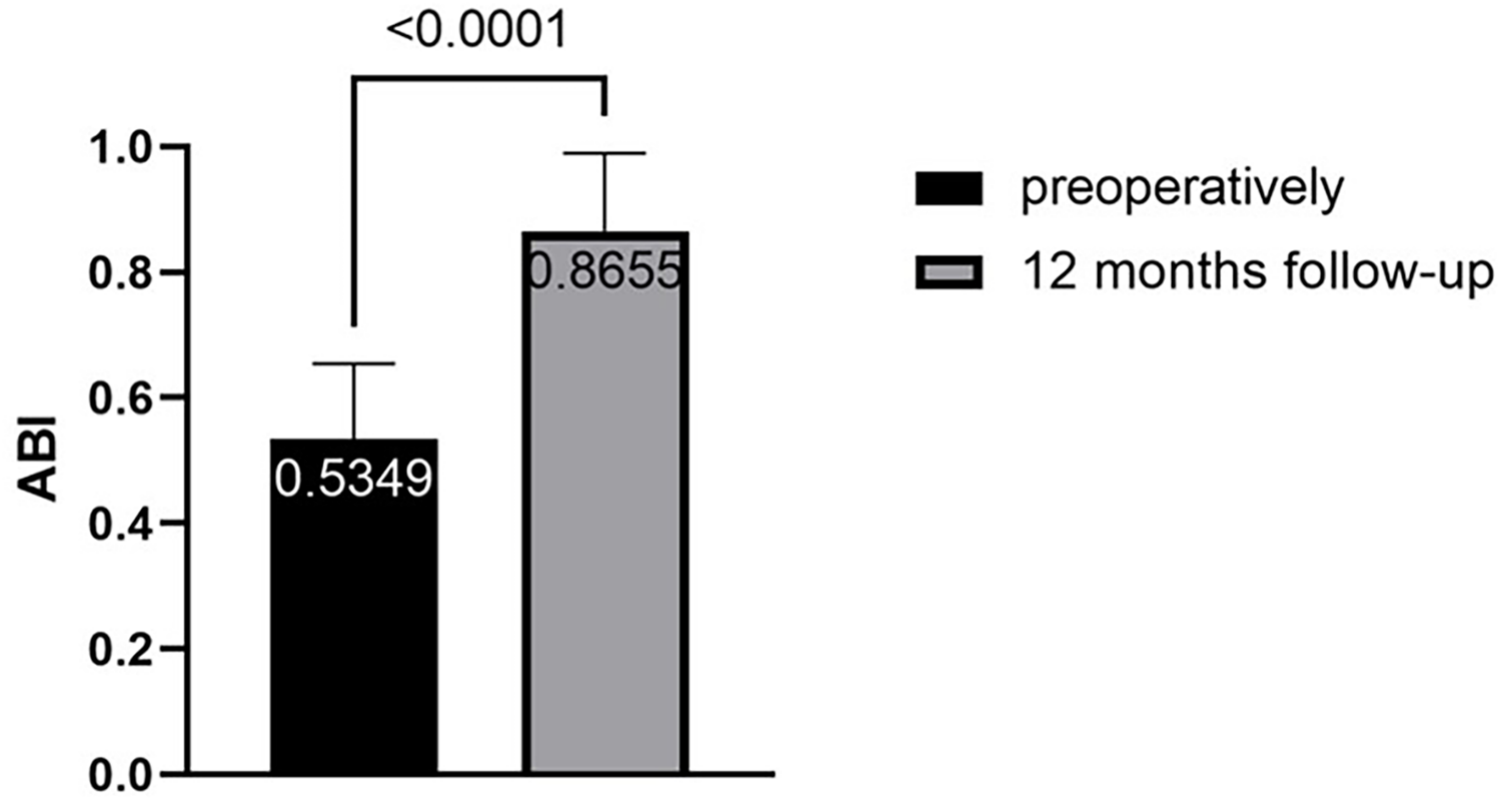
Improvement of the ABI.

**Figure 4:**
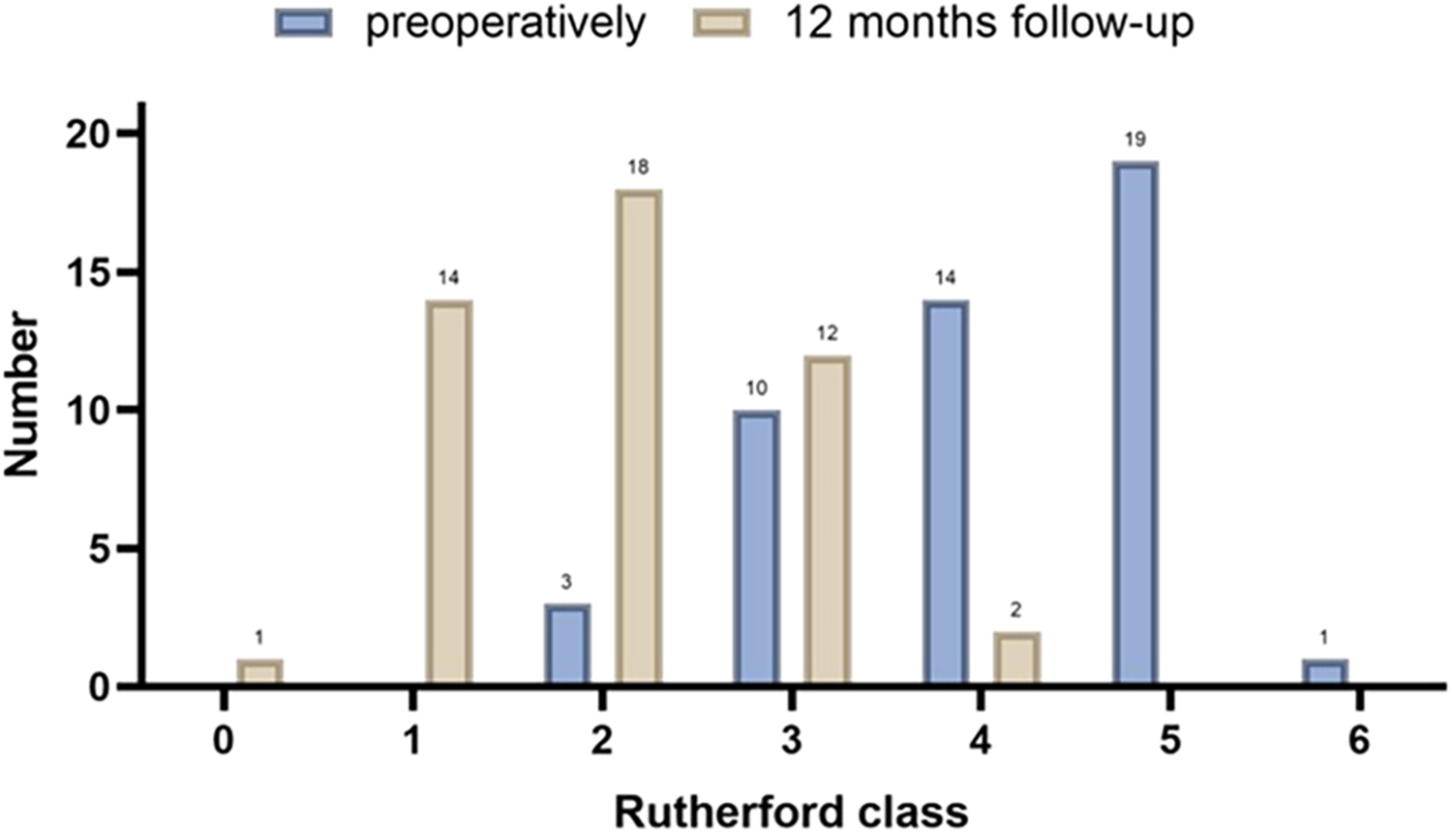
Improvement of the Rutherford classification.

All patients achieved procedural success and clinical success. The mean ABI increased from 0.53 ± 0.12 before the procedure to 0.87 ± 0.12 at 12-month follow-up, and the difference was statistically significant (*p* < 0.001, *N* = 47) (Figure 3). At 12 months, 91.5% of patients (43/47) showed a reduction in Rutherford classification. The proportion of patients with Rutherford class2 was 6.4% (3/47) at baseline, while the proportion of patients with Rutherford class 0–2 increased to 70.2% (33/47) at 12 months. The proportion of patients with Rutherford class 4–6 decreased from 70.2% (33/47) to 4.3% (2/47) at 12 months, and the difference was statistically significant (*p* < 0.001, *N* = 47) (Figure 4). Intraoperative dissection occurred in 34 limbs (72.3%), with 29 cases classified as type B or lower dissections and 5 cases classified as type C or higher (severe dissections) (10.6%). Two limbs (4.3%) required the use of salvage stents.

### Safety outcomes

At 12 months of follow-up, three patients died (one case of gastric malignancy and two cases of novel coronavirus infection), all of which were unrelated to the device or procedure. Twelve limbs (25.5%) underwent minor toe amputation. All these cases were from patients with pre-existing tissue loss (Rutherford 5–6) and were planned procedures due to pre-existing gangrene or severe tissue loss. None of the amputations were related to procedural complications or dissections. There were no occurrences of acute arterial thrombosis or puncture site bleeding in any of the patients postoperatively.

## Discussion

As mentioned earlier, the majority of PAD patients have lesions in the femoropopliteal artery or the popliteal artery, which is closely related to the anatomical position of these arteries. The femoral artery and the popliteal artery run between the muscles of the thigh, and therefore their proximal and distal segments are subject to unique external physical forces and continuous motion. The femoropopliteal artery, in particular, passes relatively superficially through the lower limb, interacting with surrounding muscle tissues and experiencing compression and torsional forces. These anatomical relationships not only contribute to femoropopliteal artery lesions being the most common vascular abnormalities in PAD patients but also influence the outcomes of endovascular interventions. Consequently, the femoropopliteal segment remains the most challenging area for the development of restenosis and reocclusion following endovascular treatments (10, 12, 13). Currently, there is no research indicating which treatment modality, either used alone or in combination, can improve the treatment outcomes of SFA and popliteal artery (PA) lesions, especially in long-segment lesions. Therefore, identifying the optimal treatment option has become a hot topic in the field of vascular surgery.

POBA is performed by inflating the balloon using a pressure pump. The balloon dilation causes focal tears in the intimal and medial layers of the narrowed vessel wall, aiming to expand the stenotic or occluded artery. Due to its simplicity, affordability, and widespread availability, PTA is highly favored by vascular surgeons and patients. It is the most commonly used method for endovascular treatment of lower extremity arterial occlusive disease. However, the efficacy of treating femoropopliteal artery lesions is not ideal due to characteristics such as elastic recoil, severe flow-limiting dissections, neointimal hyperplasia, and high restenosis rates (5, 14). Therefore, vascular surgeons have addressed the issue of improving the patency rates in the endovascular treatment of femoropopliteal artery lesions by employing the method of POBA followed by bare metal stent (BMS) placement (POBA + BMS). The VIASTAR trial demonstrated a primary patency rate of 53.5% and an f-TLR rate of 77.0% at 12 months (15). This treatment approach has shown a high rate of procedural success and reliable medium- to long-term target lesion patency. However, a significant clinical concern associated with this approach is the occurrence of in-stent restenosis (ISR), with reported rates of up to 30% and 50% at 12 and 24 months postoperatively, respectively (16, 17). Once ISR occurs, further intervention procedures are required to treat and reopen the narrowed or occluded vessel lumen. Therefore, in order to address the aforementioned issues and improve postoperative patency rates in diseased vessels, DCB have emerged as a solution. The surface of a DCB is coated with anti-restenotic drugs (such as rapamycin, paclitaxel, and their derivatives). During balloon dilation, these drugs on the surface of the drug-coated balloon effectively and continuously diffuse and adhere to the vessel wall. By stabilizing intracellular microtubules and inhibiting the mitosis of smooth muscle cells in the vessel wall, they effectively prevent neointimal hyperplasia and reduce the occurrence of in-stent restenosis(ISR) (18). A study from China showed that DCB treatment had higher patency rate (74.5% vs. 52.4%) and f-TLR (78.2% vs. 59.5%) compared to mitinol stent at 12 months (19).

Although DCB has shown promising efficacy in the treatment of femoropopliteal artery lesions, both regular balloon angioplasty and DCB can lead to vascular trauma caused by uneven balloon expansion, resulting in radial, torsional, and longitudinal stress. This trauma increases the occurrence of dissections, elastic recoil, and acute vessel occlusion, thereby raising the need for salvage stent placement (20). The incidence of dissection in this study was 72.3%, which is similar to the 79.7% reported in the recent AcoArt I study (21). This can be attributed to the higher proportion of chronic total occlusion (CTO) lesions (63.8%), longer lesion length (104.13 ± 61.12 mm), and higher degree of severe calcification (51.1%) in the cases included in our study. In order to avoid these related injuries caused by PTA, vascular surgeons have proposed different strategies for appropriate vascular preparation prior to DCB, such as intraluminal plaque excision, use of non-compliant balloon for vessel dilation, or the use of chocolate balloon angioplasty (10). The Chocolate Balloon is a nickel-titanium-constrained balloon that increases the contact area with the vessel during dilation, thus minimizing overall vascular trauma. Although the incidence of dissection in our study was relatively high, we classified the dissections that occurred during the operation by using multi-angle angiography and observation of flow velocity. We found that severe dissections (Type C or above) occurred in only 5 cases, accounting for 10.6% of the cases, and the rate of rescue stent placement was only 4.3%, much lower than that of POBA. This can be attributed to the use of the Chocolate balloon for vessel preparation during the procedure. However, it should be noted that most international researchers recommend the use of intravascular ultrasound (IVUS) for classifying dissections (22). Due to cost and medical insurance issues, this technology is not widely available in China, and only a few centers currently have access to this device.

The primary patency rate at 6 months was 87.2%, at 12 months was 78.7%, and the f-TLR rate at 12 months was 85.1% in our study, which was significantly better than using DCB alone (8). At the 12-month follow-up, we observed an improvement in Rutherford classification in 91.5% of patients. The proportion of patients in Rutherford grades 4–6 decreased from 70.2% to 4.3%, which is a highly encouraging result. For these patients, endovascular treatment is more important for improving their quality of life and achieving a favorable long-term prognosis rather than being a life-saving or limb-saving procedure (23). It is worth noting that in our study, 25.5% of patients underwent minor toe amputations, which can be attributed to the higher preoperative Rutherford class (42.5% were R5 or higher) in our enrolled patients.

Our study provides a potential method to improve long-term patency and reduce intraoperative salvage stenting in patients with femoropopliteal artery disease. At present, there are several ongoing studies exploring this aspect, such as the Chocolate Touch DCB (24) and Drug-Eluting Stent (DES) (25), which are potential methods that may achieve this goal.

This study has several limitations. Firstly, it is a single-center retrospective study. Additionally, the sample size of this study is small, and the follow-up period is short, which may introduce bias in the research findings. In the future, increasing the sample size and extending the follow-up duration will be necessary to obtain more robust research results.

## Conclusion

In summary, the combination of DCB and Chocolate PTA balloon catheter for the treatment of femoropopliteal artery disease has achieved satisfactory patency rates and f-TLR outcomes at 1-year follow-up. However, further confirmation of these findings is required through multicenter data and long-term follow-up results.

## Data Availability

The raw data supporting the conclusions of this article will be made available by the authors, without undue reservation.
